# Heterogeneity in the Metastatic Microenvironment: JunB-Expressing Microglia Cells as Potential Drivers of Melanoma Brain Metastasis Progression

**DOI:** 10.3390/cancers15204979

**Published:** 2023-10-13

**Authors:** Orit Adir, Orit Sagi-Assif, Tsipi Meshel, Shlomit Ben-Menachem, Metsada Pasmanik-Chor, Dave S. B. Hoon, Isaac P. Witz, Sivan Izraely

**Affiliations:** 1 The Shmunis School of Biomedicine and Cancer Research, The George S. Wise Faculty of Life Science, Tel Aviv University, Tel Aviv 6997801, Israel; oritadir@mail.tau.ac.il (O.A.); oritsa@tauex.tau.ac.il (O.S.-A.); tzipi@tauex.tau.ac.il (T.M.); benmena@tauex.tau.ac.il (S.B.-M.); isaacw@tauex.tau.ac.il (I.P.W.); 2 Bioinformatics Unit, The George S. Wise Faculty of Life Science, Tel Aviv University, Tel Aviv 6997801, Israel; metsada@tauex.tau.ac.il; 3 Department of Translational Molecular Medicine, Saint John’s Cancer Institute, Providence Saint John’s Health Center, Santa Monica, CA 90404, USA; dave.hoon@providence.org

**Keywords:** melanoma, brain metastasis, microglia, tumor microenvironment, immune microenvironment, JunB

## Abstract

**Simple Summary:**

Brain metastasis is a devastating but common consequence of advanced stage melanomas. The interactions between cancer cells and non-cancerous cells in the tumor microenvironment alleviate cancer progression. Previously, we found that brain metastatic melanoma cells reprogrammed microglia into tumor-promoting cells, but the mechanism is yet to be clarified. Here, we identified the transcription factor JunB to be dramatically upregulated in microglia following their exposure to melanoma. We recognized a diverse range (high and low) of JunB expression levels in melanoma-associated microglia, and therefore sought to establish two different microglia populations, expressing high or low levels of JunB, and to delineate their involvement in melanoma brain metastasis progression. High-JunB microglia demonstrated pro-tumor properties, while low-JunB microglia demonstrated anti-tumor properties. Moreover, we described the mechanism by which JunB upregulation is induced by melanoma cells. Microglia highly expressing JunB may serve as a target for brain-metastasizing melanoma therapy.

**Abstract:**

Reciprocal signaling between melanoma brain metastatic (MBM) cells and microglia reprograms the phenotype of both interaction partners, including upregulation of the transcription factor JunB in microglia. Here, we aimed to elucidate the impact of microglial JunB upregulation on MBM progression. For molecular profiling, we employed RNA-seq and reverse-phase protein array (RPPA). To test microglial JunB functions, we generated microglia variants stably overexpressing JunB (JunB^hi^) or with downregulated levels of JunB (JunB^lo^). Melanoma-derived factors, namely leukemia inhibitory factor (LIF), controlled JunB upregulation through Janus kinase (JAK)/signal transducer and activator of transcription 3 (STAT3) signaling. The expression levels of JunB in melanoma-associated microglia were heterogeneous. Flow cytometry analysis revealed the existence of basal-level JunB-expressing microglia alongside microglia highly expressing JunB. Proteomic profiling revealed a differential protein expression in JunB^hi^ and JunB^lo^ cells, namely the expression of microglia activation markers Iba-1 and CD150, and the immunosuppressive molecules SOCS3 and PD-L1. Functionally, JunB^hi^ microglia displayed decreased migratory capacity and phagocytic activity. JunB^lo^ microglia reduced melanoma proliferation and migration, while JunB^hi^ microglia preserved the ability of melanoma cells to proliferate in three-dimensional co-cultures, that was abrogated by targeting leukemia inhibitory factor receptor (LIFR) in control microglia–melanoma spheroids. Altogether, these data highlight a melanoma-mediated heterogenous effect on microglial JunB expression, dictating the nature of their functional involvement in MBM progression. Targeting microglia highly expressing JunB may potentially be utilized for MBM theranostics.

## 1. Introduction

Melanoma incidence is rising yearly worldwide. Alongside the advance in new treatment options, brain metastases frequently develop in melanoma patients. Melanoma has one of the highest propensities to metastasize to the brain compared to other cancers, with 40–60% of melanoma patients developing brain metastases. At post-mortem dissections, central nervous system (CNS) involvement is identified in up to 80% of patients with metastatic melanoma. In several tumor types, including melanoma, CNS metastases often present unique features compared to those seen in primary tumors or in metastases to other organs in the same individual. This tumor-heterogeneity and the relatively low penetration of most drugs through the BBB make CNS metastasis a frequent cause of treatment failure in melanoma patients treated with targeted therapies [1].

Based on the concept that interactions between cancer cells and non-cancerous cells in the tumor microenvironment (TME) are pivotal in cancer progression towards metastasis [2], our laboratory focuses on the interactions between melanoma brain-metastasizing (MBM) cells and brain cells such as brain endothelial cells [3], astrocytes [4], and microglia [5]. We found that the crosstalk between melanoma and microglia reprograms the phenotype and molecular signature of microglia cells [5]. For example, factors secreted by MBM cells, namely IL-6, induced the STAT3 signaling pathway and increased the expression of suppressor of cytokine signaling 3 (SOCS3) in microglia cells [6]. Inhibition of the JAK/STAT pathway resulted in reduced pro-tumorigenic functions of microglia.

Studies demonstrate a role for JAK-STAT signaling in promoting MBM [1]. For example, the JAK/STAT pathway is involved in brain neoangiogenesis, contributing to melanoma cells’ nutrient supply. Other studies showed that unlike its activation status in primary tumor tissues, STAT3 is continuously activated in brain metastasis [7]. In addition to the JAK-STAT pathway, increased activation of the PI3K-AKT pathway was identified in MBM samples compared with extracranial metastases from the same patients, at least partially as a result of transmission of PTEN-targeted microRNAs (miRNAs) delivered by astrocyte-derived exosomes [1].

In the current study, RNA-sequencing (RNA-seq) analysis showed that treating human microglia cells with soluble factors derived from MBM upregulated the expression of the transcription factor JunB in microglia. JunB is a component of the activator protein-1 (AP-1) dimeric transcription factor, which is composed of homodimers or heterodimers of proteins that belong to the Jun, Fos, and activating transcription factors (ATF) subfamilies [8].

JunB-containing AP-1 complexes regulate the transcription of a variety of genes, including: the anti-apoptotic gene Bcl-2, cell cycle genes p16INK4α cyclin-dependent kinase inhibitor and cyclin A2, regulatory T (Treg)-inducer IL-4, VEGF, MMPs, and others [9,10,11,12], thereby instigating various physiological and pathological processes.

JunB promotes the differentiation of several T-cell types [11,13] and is a dual regulator of the cell cycle [9]. In normal tissues, it inhibits proliferation and its inactivation primes reduction of apoptosis by Bcl-2 regulation [10,12]. Alternatively, JunB promotes cell division, bone marrow cell proliferation, and proliferation of embryonic fibroblasts [9,10]. JunB mediates the progression of several tumors, including chronic myelogenous leukemia (CML), different lymphomas, and carcinomas [9,14,15].

Further analysis of melanoma-exposed microglia recognized the existence of microglia cells with diverse (high or low) expression of JunB. Current research utilizes novel strategies of single-cell profiling (i.e., single-cell RNA-seq) that permit the dissection of the cellular components in the TME into subtype-specific, molecularly distinct populations, revealing the complex heterogeneity of immune cell populations [13]. The plasticity of resident and infiltrating immune cells within the TME is demonstrated in several malignancies, including brain malignancies, and in other brain pathologies [16,17,18]. This heterogeneity is shaped not only by tumor-derived signals, but may also be influenced by the tumor micromilieu, giving rise to molecularly and functionally different cell populations with either pro- or anti-tumor effects [17].

These expression pattern differences allow a deeper understanding of the heterogeneous subpopulations of tumor-associated macrophages/microglia [19].

Although single-cell profiling of immune cells in tumors indicates that most tumor-associated microglia/macrophages (TAMs) are of the pro-tumor, anti-inflammatory, alternatively activated M2 type, TAMs could also be polarized into tumor-suppressive, pro-inflammatory, M1-type cells. These M1-type TAMs can stimulate and recruit T-cells to the tumor site, thereby facilitating an inflammatory TME [19]. Single-cell RNA-sequencing analysis described by Chen et al. [18] identified a cell population where JunB is a central gene regulating the immunosuppressive, non-inflamed TME, including TAMs, in bladder cancer [13], but functional data on microglia involving JunB are still lacking to date.

The aim of the present study was to elucidate and characterize the impact of high-level JunB-expressing microglia vs. low-level JunB-expressing microglia on the malignant phenotype of MBM cells.

## 2. Materials and Methods

### 2.1. Cell Culture

Melanoma brain metastasis (MBM) variant YDFR.CB3 was established from the human melanoma brain metastasis cell line YDFR (kindly provided by Prof. Michael Micksche, Department of Applied and Experimental Oncology, Vienna University, Austria). DP.CB2, M12.CB3, and M16.CB3 MBM variants were established from human melanoma brain metastasis cell lines DP-0574-Me, UCLA-SO-M12, and UCLA-SO-M16 (kindly provided by Dave S.B. Hoon) [19,20]. Melanoma cells were maintained in 10% fetal calf serum (FCS), 2 mmol/mL L-glutamine, 100 units/mL penicillin, 0.1 mg/mL streptomycin, and 12.5 units/mL nystatin-supplemented RPMI 1640 medium (Biological Industries, Kibbutz Beit Haemek, Israel). Immortalized human microglia SV40 cells (ABM, Milton, ON, Canada) were maintained on 100 μg/mL collagen I, rat tail (Corning, Bedford, MA, USA) in Prigrow III medium (ABM) supplemented with 10% FCS, 2 mmol/mL L-glutamine, 100 units/mL penicillin, 0.1 mg/mL streptomycin, and 0.00025 units/mL amphotericin B. The human embryonic kidney 293T cell line was maintained in DMEM supplemented with 10% FCS, 2 mmol/mL L-glutamine, 100 units/mL penicillin, 0.1 mg/mL streptomycin, and 12.5 units/mL nystatin.

A mixture of RPMI 1640 and Prigrow III culture media (1:1) supplemented with 0.5% FCS was used for all experiments with starvation medium. Since microglia cells are more sensitive to serum-free conditions than melanoma cells, we used media containing 0.5% FCS for all experiments conducted with starvation medium. Cells were routinely cultured in an incubator with humidified air with 5% CO_2_ at 37 °C.

To produce GFP-expressing microglia cells, cells were transduced with a pQCXIN-GFP plasmid (Clontech Laboratories, Inc., Mountain View, CA, USA) and to produce mCherry expressing MBM cells, cells were transduced with a pQCXIP-mCherry plasmid (Clontech Laboratories, Inc.), as previously described [3,20].

### 2.2. Preparation of Melanoma- or Microglia-Conditioned Medium

Microglia or melanoma cells were cultured for 24 h, then starved in 0.5% FCS-containing medium for 24 h or 72 h, respectively. Microglia or melanoma-conditioned medium (CM) was collected, centrifuged for 5 min at 220× *g*, and filtered (0.45 μm, Whatman GmbH, Dassel, Germany).

### 2.3. Cytokines

Microglia cells were treated for 1 h with baricitinib or DMSO, then treated with cytokines for an additional 3 h. The following cytokines were used: leukemia inhibitory factor (LIF) (25 ng/mL), oncostatin M (OSM) (50 ng/mL), IL-4 (20 ng/mL), IL-6 (20 ng/mL), IL-15 (50 ng/mL), and IL-27 (20 ng/mL). All cytokines were purchased from PeproTeck (Rocky Hill, NJ, USA).

### 2.4. Animals

Male athymic nude mice (BALB/c background) were maintained as described in previous studies [21] under the regulations and standards of the Tel Aviv University Institutional Animal Care and Use Committee (ethical approval code: 04-19-073) and were used when they were 7–8 weeks old.

### 2.5. Intracardiac Inoculation of Tumor Cells and Immunostaining

To generate brain metastasis, mice were inoculated intracardially as previously described [6]. Six weeks following the inoculation, mice were perfused with 4% paraformaldehyde, sacrificed, and brains were fixed and embedded in optimal cutting temperature compound (Tissue-Tek^®^ O.C.T., Sakura Finetek USA, Inc., Torrance, CA, USA).

OCT-embedded 10 µm brain sections were cut. The sections were blocked for 30 min. with CAS-Block^TM^ solution (008120, Life Technologies, Carlsbad, CA, USA). Primary antibodies (Abs) were incubated in Ab diluent (ab64211, Abcam, Cambridge, UK) with 0.02% Triton X-100 overnight at 4 °C, followed by fluorescently conjugated secondary Abs in 0.02% Triton X-100 in PBS for 60 min at RT. Coverslips were mounted using 4′,6-diamidino-2-phenylindole (DAPI) Fluoromount (Southern Biotech, Birmingham, AL, USA). The images were acquired with a × 40/1.10 water objective, using a Leica SP8 confocal microscope and Leica SP8 software (LAS-X v3.5.7.23225, Leica Microsystems, Wetzlar, Germany).

Information on primary Abs against JunB, Iba-1, and melanoma markers is detailed in Table S1.

### 2.6. RNA Preparation and Reverse Transcription Quantitative Real-Time PCR (RT-qPCR)

Total cellular RNA was extracted as previously described [6]. Primer sequences are detailed below: SOCS3: S-5′-CCATTCGGGAGTTCCTGGAC-3′, AS-5′-TTGGCTTCTTGTGCTTGTGC-3′; SERPINE1: S-5′-CAACCCCACAGGAACAGTCC-3′, S-5′-TTTGTCCCAGATGAAGGCGT-3′; ALDOC: S-5′-TGCCTATTGTGGAACCTGAA-3′, AS-5′-ACAGCAGCCAAGACCTTCTC-3′; NQO1: S-5′-GGACTGCACCAGAGCCAT-3′, AS-5′-GCCTCCTTCATGGCATAGTT-3′; RS9: S-5′-CGGAGACCCTTCGAGAAATCT-3′, AS-5′-GCCCATACTCGCCGATCA-3′.

Quantification of mRNAs was normalized to the expression of RS9 as a reference gene using the Bio-Rad CFX Maestro 2.0 software v5.0.021.0616 (Bio-Rad Laboratories, Hercules, CA, USA).

### 2.7. Western Blotting

For protein expression analysis in microglia, microglia cells were starved with a mixture of RPMI 1640 and Prigrow III culture media (1:1) supplemented with 0.5% FCS for 1 h prior to treatment with melanoma-conditioned medium (MCM) or with recombinant cytokines. For experiments employing signaling pathway inhibitors or LIF receptor (LIFR) inhibitor, EC359 (HY-120142, MedChemExpress, Monmouth Junction, NJ, USA), microglia cells were starved with the relevant inhibitor (diluted in DMSO) or with DMSO, as control, for 1 h, prior to treatment with MCM.

Protein detection by Western blot was performed as previously described [6]. Primary Abs against the following proteins were used: JunB, phospho-STAT3 (Tyr705), STAT3, OSM, and anti-β-tubulin (loading control) (Table S1). Horseradish peroxidase-conjugated goat anti-mouse or goat anti-rabbit (1:10,000, Jackson ImmunoResearch Laboratories, West Grove, PA, USA) was used as secondary Abs. The bands were visualized by chemiluminescence ECL reactions (Merck Millipore, Darmstadt, Germany), and band density was quantified by Quantity One^®^ software v4.6.6 (Bio-Rad Laboratories).

### 2.8. ELISA Assay

For the estimation of secreted LIF levels, MBM cells were plated and grown in starvation media composed of a mixture of RPMI 1640 and Prigrow III culture media (1:1), supplemented with 0.5% FCS, for 72 h. The supernatants were then collected, centrifuged, filtered, and 30-fold concentrated at 4000× *g* using Amicon^®^ Ultra-15 centrifugal filter units (Merck Millipore, Darmstadt, Germany) for 1 h. The fraction (MW > 3 kDa) was used to determine the extracellular levels of LIF by ELISA according to the manufacturer’s instructions using the Human LIF DuoSet ELISA Development Kit (DY7734-05, R&D Systems, Minneapolis, MA, USA).

For the estimation of intracellular OSM levels, MBM cells were plated and grown in starvation media for 72 h. The cells were then lysed as previously described [20], and 2 µg of the lysates was used to determine the intracellular levels of OSM using the Human OSM DuoSet ELISA Development Kit (DY295, R&D Systems).

### 2.9. Flow Cytometry

The following antibodies were used for detection by flow cytometry as previously described [22]: LIFR, gp130, PD-L1, and detection of activation markers was performed similarly using the conjugated antibodies CD14-APC, CD16-APC, CD150-APC, and CD163-APC (Table S1).

For the detection of intracellular JunB and Iba-1, microglia cells were fixed with methanol for 20 min at −20 °C, then washed twice with RPMI 1640 supplemented with 10% FCS, and processed as previously described [17].

Antigen expression was determined using an S100EXi flow cytometer (Stratedigm, Inc., San Jose, CA, USA) with CellCapTure software v4.1 (https://stratedigm.com/cellcapture/ accessed on 5 February 2023) (Stratedigm, Inc.) and FlowJo v10 (FlowJo, Ashland, OR, USA). Dead cells were gated out from the analysis.

### 2.10. Construction of JunB^hi^ and JunB^lo^ Microglia Cells

The JunB^hi^ microglia cells overexpressing the human JunB (NM_002229) were established by PCR amplification of genomic DNA by KAPA HiFi DNA Polymerase (Kapa biosystems, Wilmington, MA, USA), using the following primers (designed based on the GenBank Nucleotide Database of the NCBI website): JunB: S-5′-ATATAGTCCGGTATGTGCACTAAAATGGAACAGCC-3′, AS-5′-ATTATTGGATCCGTTCAGAAGGCGTGTCCCTT-3′. The generated fragment was digested with *Age1* and *BamH1* and ligated into the corresponding sites of the pQCXIP vector (Clontech Laboratories, Inc.). PCR products of JunB were sequenced and found to be identical to the published sequence. An empty pQCXIP vector was used as a negative control. These variants are referred as MG con^hi^ and MG JunB^hi^.

The JunB^lo^ microglia cells were established by using a mixture of three different pGIPZ vectors containing shRNA sequences targeting JunB mRNA (RHS4430-200225817, RHS4430-200229510, and RHS4430-200246702; Dharmacon, Lafayette, CO, USA). A sh-non-silencing pGIPZ vector (RHS4531, Dharmacon) was used as a negative control. The variants are referred as MG con^lo^, MG JunB^lo^.

The production of plasmids and viral vectors and microglia and melanoma transduction were performed as previously described [3,20].

### 2.11. RNA-Sequencing Analysis

MCM prepared from two MBM cell lines (DP.CB2 and M12.CB3) or starvation medium were added to microglia cells for 3 h. RNA extraction and sequencing were performed as previously described [6].

Raw RNA-seq reads were checked for overall quality and filtered for adapter contamination using Trimmomatic (version 0.36) [23]. The filtered reads were then mapped to the GENCODE comprehensive gene annotation reference set (version 19) using the STAR aligner (version 2.4.2a) [24] with default parameters. Read counts for each feature were generated using the “—quantModeGeneCounts” function in STAR. Significantly differentially expressed genes (DEGs) were identified using ANOVA with a significance cutoff pAdj < 0.05 and fold change (FC) FC ≤ −2 or FC ≥ 2.

Enriched biological processes of the differentially expressed genes were obtained through the Database for Annotation, Visualization and Integrated Discovery (DAVID) [25,26].

The Venny tool v2.1 (https://bioinfogp.cnb.csic.es/tools/venny/index.html, accessed on 26 July 2022) was used to compare between differentially expressed gene lists.

### 2.12. Reverse-Phase Protein Analysis (RPPA)

Protein lysates from microglia cells were extracted as previously described [20]. RPPA was performed by the RPPA Core Facility at the University of Texas, MD Anderson Cancer Center (Michael A. Davies). As a result, 492 proteins were identified and expression data analyzed using Partek Genomics Suite (PGS7.20.0831). Differentially expressed proteins were obtained with cutoff *p* < 0.05 and fold-change difference = 1.25.

### 2.13. Inhibitors

Microglia cells were starved for 1 h with signaling pathway inhibitors or with DMSO as control, then treated with MCM and either the inhibitor or DMSO for 3 h. The following inhibitors were used: zoledronic acid (1 µM, ALX-430-153, Enzo Life Sciences, Inc., Farmingdale, NY, USA), baricitinib (2 µM, A892931, Amadis Chemical, Hangzhou, Zhejiang, China), SP600125 (20 µM, S5567, Sigma-Aldrich, St. Louis, MO, USA), and U0126 (5 µM, CST-9903S, Cell Signaling Technology, Danvers, MA, USA). Lysates were prepared as previously described [20].

### 2.14. Viability Assay (XTT)

First, 6 × 10^3^ microglia cells were seeded on a collagen-coated 96-well plate for 24–72 h. Alternatively, 5 × 10^3^ MBM cells were seeded on a 96-well plate for 24 h, then treated with microglia CM for 48 h. Cell viability was determined using a Cell Proliferation Kit (XTT, Biological Industries). Cell viability at 24 h after seeding was determined as time point 0. To obtain the relative cell viability, the optical density (OD) of each cell variant (at each time point) was divided by the OD of the corresponding variant at time point 0.

### 2.15. Apoptosis Assay

First, 5 × 10^4^ MBM cells were seeded on a 96-well plate for 24 h, then treated with CM of microglia cells for 24 or 48 h. Quantification of apoptotic and necrotic cells was performed by annexin V and propidium iodide staining with the MEBCYTO^®^ Apoptosis Kit (MBL International, Woburn, MA, USA) according to the manufacturer’s instructions.

### 2.16. Spheroid (3D) Cultures

First, 1 × 10^3^ MBM cells and 1 × 10^3^ microglia cells in starvation medium, with EC3596 or DMSO, were seeded into 96-well low-attachment U-shaped plates (Greiner Bio-One, Frickenhausen, Germany) and centrifuged for 5 min at 1000 rpm. The wells were imaged every 6 h for 48 h, and spheroid parameters were analyzed using the IncuCyte system (Essen BioScience, Inc., Ann Arbor, MI, USA).

### 2.17. Wound-Healing Assay

Microglia cells were seeded on a collagen-coated 96-well plate. On confluence, the cell monolayer was scratched using a 96-well WoundMaker (Essen BioScience, Inc.). Cells were washed twice and growth medium was applied on the cells (a mixture of RPMI 1640 and Prigrow III culture media (1:1) supplemented with 10% FCS) for 48 h.

MBM cells were seeded on a collagen-coated 96-well plate. On confluence, the cells were treated with mitomycin C (10 µg/mL; M0503, Sigma-Aldrich) for 3 h, after which the cell monolayer was scratched using a 96-well WoundMaker. Cells were washed twice and microglia-conditioned medium (CM) or starvation medium was applied on the cells for 48 h.

The wounds were imaged every 3 h for 48 h, and images were analyzed using the IncuCyte S3 system (Essen BioScience, Inc.). Each experiment was repeated at least three times.

### 2.18. Phagocytosis Assay

First, 6.2 × 10^4^ microglia cells were seeded on a collagen-coated 24-well plate for 24 h. Cells were then starved for 1 h, after which 5 µL of opsonized beads were added to each well for 4 h. The cells were washed three times with PBS, trypsinized and centrifuged, and then fixed with cold methanol for 20 min. Following two washes with 10% FCS-supplemented RPMI 1640, bead uptake was measured by flow cytometry as described above.

Fluorescent latex beads (L2778, Sigma-Aldrich) were opsonized with RPMI 1640 supplemented with 10% FCS (1:10) for 1 h at 37 °C.

### 2.19. Nitric Oxide (NO) Production

First, 3 × 10^4^ microglia cells were seeded on a collagen-coated 96-well plate. The cells were then starved for 1 h, after which they were treated with 30-fold concentrated MCM for 24 h. Concentrated MCM was applied on empty wells as control. NO levels in the medium were measured using the Griess reagent (Promega Corporation, Madison, WI, USA) [27] according to the manufacturer’s protocol. Briefly, 50 μL of supernatant was added to a 96-well plate, followed by 50 μL sulfanilamide and 50 μL N-1-napthylethylenediamine dihydrochloride (NED). Absorbance at 540 nm was measured by a microplate reader and nitrite concentrations were estimated using a standard nitrite curve. The nitrite in MCM-treated microglia supernatant was divided by that of the corresponding MCM. At least 3 replicates were used in each experiment.

### 2.20. Biostatistic Analysis

Data were analyzed using Student’s *t*-test and considered significant at *p*-values < 0.05. Bar graphs represent mean and standard error of the mean (SEM) across multiple independent experimental repeats. Each of the experiments performed in this study was repeated at least 3 times.

## 3. Results

### 3.1. Melanoma Impacts Microglial Molecular Expression

#### 3.1.1. Melanoma-Conditioned Medium (MCM) Alters the Gene Expression Profile of Microglia Cells

In order to explore the influence of melanoma cells on microglia, the resident macrophages of the brain, we profiled the gene expression pattern of microglia cells exposed to factors secreted by four different brain-metastasizing melanoma (MBM) cell lines generated previously in our lab [28].

RNA-seq analysis of mRNA extracted from microglia cells treated with melanoma-conditioned medium (MCM) or with starvation medium (control) for 3 or 24 h identified 621 and 1353 genes differentially expressed in microglia cells treated with DP.CB2-conditioned medium (CM) for 3 or 24 h, correspondingly, compared to the control microglia cells, and 504 and 663 genes differentially expressed in microglia cells treated with M12.CB3 CM for 3 or 24 h, correspondingly (pAdj < 0.05 and FC ≤ −2 or FC ≥ 2).

**Figure 1:**
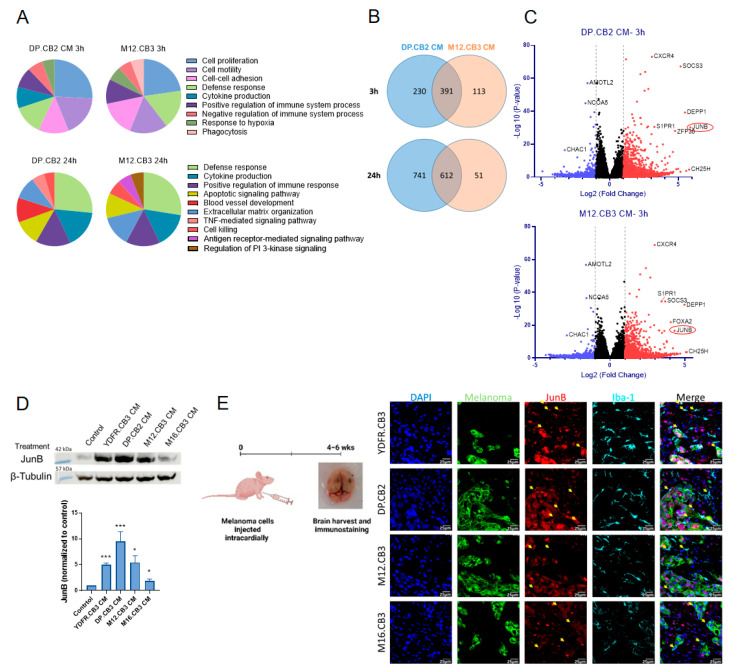
Melanoma-secreted factors reprogram gene expression of microglia. (**A**–**C**) mRNA samples of microglia cells treated with MCM for 3 or 24 h were sequenced on an Illumina NextSeq 550. (**A**) Genes differentially expressed in microglia treated with DP.CB2 CM (**left**) or M12.CB3 CM (**right**) at 3 h (**top** charts) or 24 h (**bottom** charts) (pAdj < 0.05 and FC ≤ − 2 or FC ≥ 2) were classified and selected biological processes using the DAVID database are shown. (**B**) Venn diagrams comparing genes differentially expressed in microglia treated with either DP.CB2 CM or M12.CB3 CM for 3 and 24 h. (**C**) Volcano plots showing gene expression of microglia treated with DP.CB2 or M12.CB3 CM for 3 h. Upregulated genes (pAdj < 0.05 and FC ≥ 2) are shown in red, downregulated genes (pAdj < 0.05 and FC ≤ −2) are shown in blue, and genes that did not significantly change are shown in black. Selected genes are highlighted. (**D**) Western blot analysis of JunB and β-tubulin expression in microglia cells treated with MCM for 3 h. Representative blot and quantification of JunB expression are presented. Data are shown as mean expression + SEM of biological replicates. * *p* < 0.05, *** *p* < 0.005. (**E**) YDFR.CB3, DP.CB2, M12.CB3, and M16.CB3 cells were intracardially inoculated into BALB/c nude mice, and the brains were harvested after 4–6 weeks. Brain sections were stained for melanoma (green), JunB (red), and Iba-1 (cyan). Cell nuclei were stained with DAPI (blue). Yellow arrows indicate co-expression of Iba-1 and JunB. Magnification: ×40, scale bar: 25 μm.

The DAVID database revealed that the genes altered in microglia treated with these MCM are classified into the following biological processes: cell proliferation, motility, adhesion, positive or negative regulation of immune response, and others, as detailed in Figure 1A and Table S2.

As shown by the Venn diagram (Figure 1B), 63% of the genes differentially expressed in microglia following 3 h treatment with DP.CB2 CM and 78% of the genes differentially expressed in microglia following 3 h treatment with M12.CB3 CM were common. Similarly, 45% of the genes differentially expressed in microglia following 24 h treatment with DP.CB2 CM and 92% of the genes differentially expressed in microglia following 24 h treatment with M12.CB3 CM were common. Therefore, we deduce that CM from both melanoma cell lines had, in part, a similar effect on the molecular expression of genes in microglia. However, the non-mutual differentially expressed genes highlight the existence of dissimilar effects of the secretome of melanomas from different individuals on the tumor microenvironment.

One of the genes that were significantly upregulated in microglia following 3 h of treatment with MCM from DP.CB2 or M12.CB3 cells was the transcription factor JunB, as shown in the volcano plots (Figure 1C). This finding was validated at the protein level using Western blotting of extracts from microglia cells treated with CM of four MBM cell lines. CM of YDFR.CB3, DP.CB2, M12.CB3, and M16.CB3 cells induced a significant upregulation of JunB in microglia cells, by a mean FC of 5, 9.5, 5.4, and 1.9, respectively (Figure 1D). Notably, CM of the M16.CB3 cell line had the smallest effect on JunB expression by microglia.

We next asked whether JunB is expressed by microglia and infiltrating tumor-associated macrophages (TAMs) in brains of mice with MBM. Brain sections of mice bearing metastases, formed by intracardiac inoculation of MBM cell lines YDFR.CB3, DP.CB2, M12.CB3, and M16.CB3, were stained for JunB and microglia/TAM marker Iba-1 (Figure 1E). Indeed, we identified JunB-expressing microglia/TAMs adjacent to melanoma lesions, emphasizing the significance of delineating the role of JunB in MBM-associated microglia/TAMs.

#### 3.1.2. MCM Upregulates JunB Expression in Microglia through the JAK/STAT Signaling Pathway

**Figure 2:**
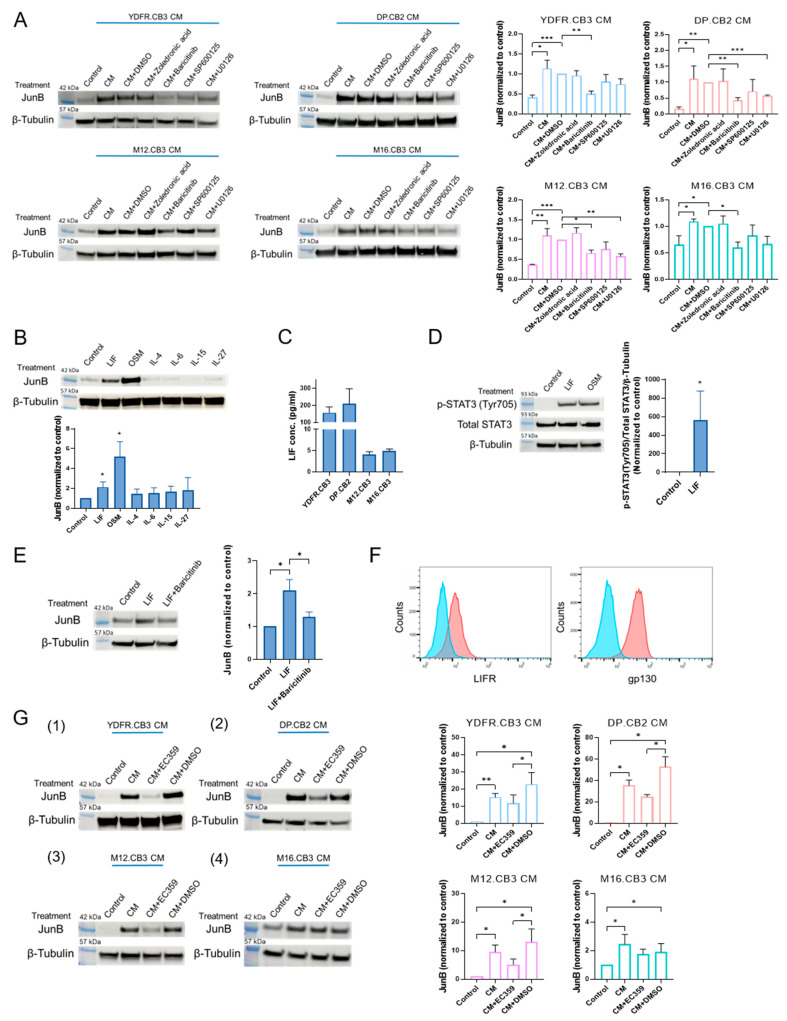
MBM-derived leukemia inhibitory factor (LIF) upregulates JunB expression in microglia via JAK/STAT3 signaling. (**A**) Western blot analysis of JunB and β-tubulin in microglia cells treated with MCM combined with STAT3 (S727) inhibitor (zoledronic acid), JAK inhibitor (baricitinib), JNK inhibitor (SP600125), MEK/ERK inhibitor (U0126), and DMSO as a control for 3 h. Representative blot and quantification of JunB expression are presented (normalized to cells treated with MCM + DMSO). Data are shown as mean expression + SEM of biological replicates. * *p* < 0.05, ** *p* < 0.01, *** *p* < 0.005. (**B**) Western blot analysis of JunB and β-tubulin expression in microglia cells treated with LIF (25 ng/mL), OSM (50 ng/mL), IL-4 (20 ng/mL), IL-6 (20 ng/mL), IL-15 (50 ng/mL), and IL-27 (20 ng/mL) for 3 h. Representative blot and quantification of JunB expression are presented. Data are shown as mean expression + SEM of biological replicates. * *p* < 0.05. (**C**) ELISA measurement of LIF in MCM. The bars represent the average cytokine concentration + SEM. (**D**) Western blot analysis of p-STAT3 (Tyr705) and STAT3 expression in microglia cells treated with LIF (25 ng/mL) for 10 min. Representative blot and quantification of p-STAT3 (Tyr705), STAT3, and β-tubulin expression are presented. Data are shown as mean expression + SEM of biological replicates. * *p* < 0.05. (**E**) Western blot analysis of JunB and β-tubulin expression in microglia cells treated with LIF (25 ng/mL) with or without baricitinib for 3 h. Representative blot and quantification of JunB expression are presented. Data are shown as mean expression + SEM of biological replicates. * *p* < 0.05. (**F**) Flow cytometry analysis of LIFR and gp130 expression in microglia cells. (**G**, 1–4) Western blot analysis of JunB and β-tubulin in microglia cells treated with or without MCM with LIFR inhibitor (EC359) or DMSO as control for 3 h. Representative blot and quantification of JunB expression are presented. Data are shown as mean expression + SEM of biological replicates. * *p* < 0.05, ** *p* < 0.01. In all the experiments, unless indicated otherwise, microglia cells grown in starvation medium served as controls.

Signaling pathway inhibitors were used to identify signaling pathways involved in MCM-mediated JunB upregulation in microglia cells. Microglia cells were treated with MCM from the four melanoma cell lines together with inhibitors for STAT3 (targeting Ser727, but not the Tyr705 residue)—which is downstream to JAK activation, JAK, JNK or MEK/ERK for 3 h. JunB protein expression in the treated and control microglia was then measured using Western blotting (Figure 2A). Baricitinib (inhibiting JAK and downstream STAT3 phosphorylation on the Tyr705 residue in microglia [6]) abrogated JunB upregulation in microglia treated with MCM from all four MBM lines. The MEK/ERK inhibitor, U0126, abrogated JunB upregulation only in microglia treated with MCM from two MBM cell lines (DP.CB2 and M12.CB3). The STAT3 (Ser727) inhibitor, zoledronic acid, and JNK inhibitor, SP600125, did not inhibit JunB upregulation in MCM-treated microglia. These results demonstrate that melanoma cells positively regulate the expression of JunB in microglia and that this upregulation is mediated mainly by JAK/STAT (through the phosphorylation of Tyr705, but not Ser727) and in some cases also by the MEK/ERK pathway.

#### 3.1.3. Leukemia Inhibitory Factor Secreted by Melanoma Cells Upregulates Microglial JunB via the JAK/STAT Signaling Pathway

In order to identify the factors in MCM that upregulate JunB in microglia, we screened six candidate cytokines, namely leukemia inhibitory factor (LIF), oncostatin M (OSM), IL-4, IL-6, IL-15, and IL-27, all known to signal through the JAK pathway [29,30,31]. Previous analysis of the secretome of MBM revealed that these cytokines are expressed by melanoma cells, and previous data obtained by RNA-seq showed that the corresponding receptor subunits are expressed on MCM-treated microglia.

Microglia cells were treated with the above recombinant cytokines for 3 h. Western blot analysis demonstrated that LIF and OSM treatment significantly upregulated JunB in microglia (Figure 2B), while the other cytokines did not.

To validate that MBM indeed produce LIF and OSM, we quantified by ELISA the levels of the two cytokines in the conditioned medium of the four MBM cell lines. YDFR.CB3 and DP.CB2 secreted higher levels of LIF (157.23 pg/mL and 210.44 pg/mL, respectively) than M12.CB3 and M16.CB3 (4.13 pg/mL and 4.84 pg/mL, respectively) (Figure 2C). OSM was not detected in the MCM, however, we did detect OSM intracellular expression in extracts of melanoma cells employing ELISA.

Following a 10 min exposure to recombinant LIF, an induction in STAT3 phosphorylation in the Tyr705 residue was observed, indicating the activation of the JAK/STAT pathway by LIF (Figure 2D). Baricitinib abolished this upregulation (Figure 2E), confirming the involvement of the JAK/STAT pathway in the regulation of JunB.

LIF acts via a heterodimeric receptor consisting of gp130 and LIF receptor (LIFR) [29]. FACS analysis of microglia cells confirmed that microglia express both LIFR and gp130 subunits (Figure 2F).

To test whether LIF is the main inducer of JunB upregulation in MCM-exposed microglia, we treated microglia with MCM together with the LIFR inhibitor, EC359, or with DMSO as control. Western blot analysis showed that EC359 indeed abrogated JunB upregulation in microglia treated with MCM of three out of four melanoma cell lines, excluding CM of M16.CB3 cells (Figure 2G, 1–4). As reported above, CM of the M16.CB3 cells had a significant, however relatively minor, effect on JunB expression by microglia.

These results demonstrate that JunB upregulation in microglia was mediated (at least partially) by melanoma-derived LIF, through the activation of LIFR.

### 3.2. Melanoma-Associated Microglia Cells Expressing High JunB Levels Exhibit a Pro-Tumorigenic, Immunosuppressive Phenotype

#### 3.2.1. Factors Secreted by Melanoma Cells Induce JunB Expression Heterogeneity in Microglial Cells

As demonstrated in Figure 1D, MCM-treated microglia cells express, on average, higher levels of JunB than untreated microglia.

A large amount of recently published data demonstrate the induction of heterogeneity in populations of tumor-associated microglia/macrophages, both at the molecular and functional levels [19,32,33].

**Figure 3:**
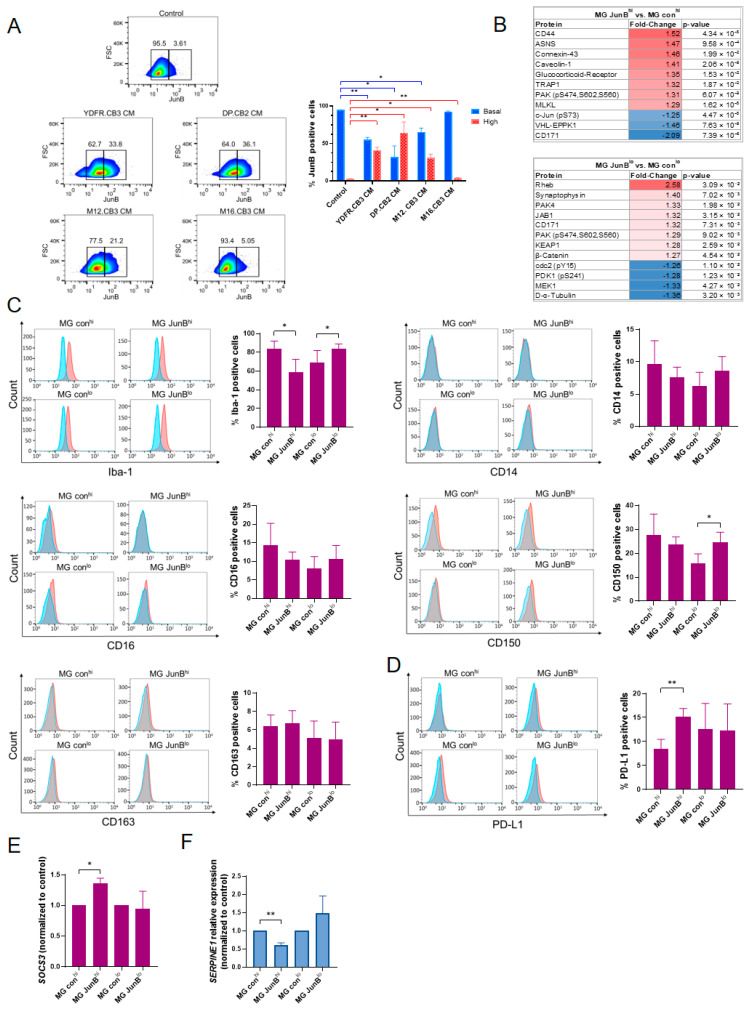
JunB regulates the molecular phenotype of microglia cells. (**A**) Flow cytometry analysis of intracellular JunB expression in microglia cells treated with MCM for 3 h. The colors used refer to the density of the cells relative to JunB expression. Blue and green correspond to lower cell density. Yellow is mid-range cell density. Red and orange correspond to higher cell density. The left rectangles mark the expression range of JunB in ~95% of control microglia and the right rectangles mark the expression range of JunB in ~5% of control microglia. The bars on the graph represent the mean % of cells expressing basal JunB levels (blue bars) and the mean % of cells expressing high JunB levels (red bars) + SEM, * *p* < 0.05, ** *p* < 0.01. (**B**) Protein lysates of JunB^hi^ and JunB^lo^ and matching control microglia cells were analyzed for the expression of 492 proteins using RPPA. The tables list the differentially expressed proteins (*p* < 0.05, FC ≤ −1.25 or FC ≥ 1.25) in JunB^hi^ (**top**) and JunB^lo^ (**bottom**) microglia cells compared to their controls. (**C**) Iba-1, CD14, CD16, CD150, and CD163 expression was determined using flow cytometry in JunB^hi^ and JunB^lo^ microglia cells and their controls. Representative flow cytometry histograms of marker expression are shown. The bars represent the mean percentage of positive cells + SEM. * *p* < 0.05. (**D**) PDL-1 expression was determined using flow cytometry in JunB^hi^ and JunB^lo^ microglia cells and their controls. Representative flow cytometry histograms are shown. The bars represent the mean percentage of positive cells + SEM. ** *p* < 0.001. (**E**,**F**) The relative expression of SOCS3 (**E**) and SERPINE1 (**F**) mRNA in JunB^hi^ and JunB^lo^ microglia cells and their controls was detected by RT-qPCR. RS9 was used for gene expression normalization. The bars represent the mean expression of SOCS3 (normalized to control cells) + SEM, * *p* < 0.05, ** *p* < 0.01.

In order to characterize how MBM cells affect JunB expression levels in microglia, microglia cells exposed to MCM were tested for intracellular JunB expression by flow cytometry. Microglia cells treated with MCM from three out of the four MBM cell lines expressed variable levels of JunB, as demonstrated by the wide distribution of cells along the histogram in Figure 3A, compared to the rather homogenous expression levels of JunB in untreated microglia (demonstrated by a narrower distribution of this protein levels). In microglia exposed to YDFR.CB3, DP.CB2, and M12.CB3 CM, 32–65% of microglia cells, on average, expressed JunB levels that are similar to those in the untreated microglia, while 31–65% of microglia cells, on average, expressed higher levels of JunB than the basal levels expressed by 95% of untreated microglia. M16.CB3 CM-treated microglia resembled the JunB expression distribution of the untreated microglia, with only 4% of cells expressing high JunB levels, compared to 2.6% in the control microglia cells; a small, although significant, change.

#### 3.2.2. Generation of Stable Microglia Variants Expressing High or Low Levels of JunB

The diversity between microglia cells expressing high or low levels of JunB may be reflected by differential phenotypes and functional capabilities of these two types of cells. In order to examine this possibility, we generated stable variants of microglia expressing either high (JunB^hi^) or low (JunB^lo^) levels of JunB. Microglia cells were infected with lentivirus containing JunB, shJunB constructs, or with mock plasmids as control (con^hi^ and con^lo^, respectively). These cellular variants were validated for their differential JunB expression (Supplementary Figure S1A).

#### 3.2.3. JunB^hi^ and JunB^lo^ Microglia Cells Differ in Their Proteomic Expression Profile

Reverse-phase protein array (RPPA) analysis [34] was performed in order to identify proteins that are either up- or downregulated in microglia cells expressing high or low levels of JunB. The expression of 492 proteins in JunB^hi^ and JunB^lo^ microglia cells was compared to that in matching control microglia cells. For each pair of samples (JunB^hi^ vs. con^hi^ and JunB^lo^ vs. con^lo^ microglia cells), we established a list of proteins that were differentially expressed by a *p* < 0.05, FC ≤ −1.25 or FC ≥ 1.25 (Figure 3B). A total of 11 and 12 proteins were differentially expressed in JunB^hi^ and JunB^lo^ microglia cells (respectively), compared to their controls. Among the significantly upregulated proteins in JunB^hi^ microglia cells we found the proteins: CD44, caveolin-1, glucocorticoid receptor, and TRAP1, all of which were found to be associated with reduced inflammatory response or resolution of inflammation in macrophages [35,36,37,38], and therefore may facilitate tumor-supporting properties in JunB^hi^ microglia. Among the significantly upregulated proteins in JunB^lo^ microglia cells we found the proteins: Rheb, synaptophysin, and KEAP1, which are reported to be associated with inflammatory response [39,40,41], and therefore may facilitate tumor-restraining properties in JunB^lo^ microglia.

#### 3.2.4. JunB^hi^ and JunB^lo^ Microglia Differentially Express Microglial Activation Markers and Immunosuppressive Molecules

To better characterize the expression of malignancy-related molecules in microglia expressing high JunB levels or low JunB levels, and since JunB modulates both pro- and anti-inflammatory phenotypes in macrophages [42], we first asked if JunB^hi^ or JunB^lo^ microglia cells differ in the expression of microglial activation markers. The expression of Iba-1, CD14, CD16, CD150, and CD163 was analyzed by flow cytometry. While no changes were observed in CD14, CD16, and CD163 expression, JunB^hi^ microglia cells expressed significantly lower levels of the pro-inflammatory Iba-1, and JunB^lo^ microglia cells expressed significantly elevated levels of Iba-1 and CD150 (Figure 3C). Iba-1 is a marker of microglia activation under an inflammatory stimulation [43]. Although considered an anti-inflammatory microglia marker, CD150-deficient macrophages secreted reduced levels of TNF-α and IL-12, indicating a pro-inflammatory activity of CD150 [44]. Thus, these results might indicate that microglia expressing high JunB levels are polarized to a more anti-inflammatory phenotype than microglia expressing low JunB levels, which are polarized, according to some indications, to a more pro-inflammatory phenotype.

MCM-derived factors upregulate the expression of the immunosuppressive molecules PD-L1 (unpublished) and suppressor of cytokine signaling 3 (SOCS3) [6] by microglia cells, both of which negatively control various immune responses [45,46,47]. In view of these results, we asked if microglia cells expressing high or low JunB levels differ in the expression of these molecules. The expression of PD-L1 as well as that of SOCS3 was significantly higher in JunB^hi^ microglia cells compared to control cells. There was no difference in the expression of these molecules in JunB^lo^ microglia cells compared to their control (Figure 3D,E). These results suggest that JunB^hi^ microglia may be associated with immunosuppression in the microenvironment of MBM.

Next, we tested the expression of the serpin family E member 1 (SERPINE1) gene, encoding the plasminogen activator inhibitor 1 (PAI-1) protein, in microglia cells expressing high or low JunB levels. PAI-1 promotes the migration and phagocytic ability of microglia cells [48]. qPCR analysis of SERPINE1 expression in JunB^hi^ and JunB^lo^ microglia cells indicated a significant decrease in SERPINE1 expression in JunB^hi^ microglia cells. However, no change was observed in the expression levels of this molecule in JunB^lo^ microglia cells (Figure 3F). These findings suggest that JunB^hi^ microglia cells may contribute to MBM progression, as their phagocytic activity and motility may be reduced.

#### 3.2.5. JunB^hi^ and JunB^lo^ Microglia Cells Differ in Their Migratory Capacity and Phagocytic Activity

High motility of macrophages has been attributed to resolution of inflammation [49], but on the other hand, macrophage migration into a damaged site plays a critical role in mediating immune defenses such as secretion of pro-inflammatory cytokines, phagocytosis, and antigen presentation [50]. In order to test if indeed microglia cells expressing high JunB levels differ in their ability to migrate and to perform phagocytic activity, we tested these two characteristics in JunB^hi^ and JunB^lo^ microglia cells vs. the corresponding control microglia cells.

**Figure 4:**
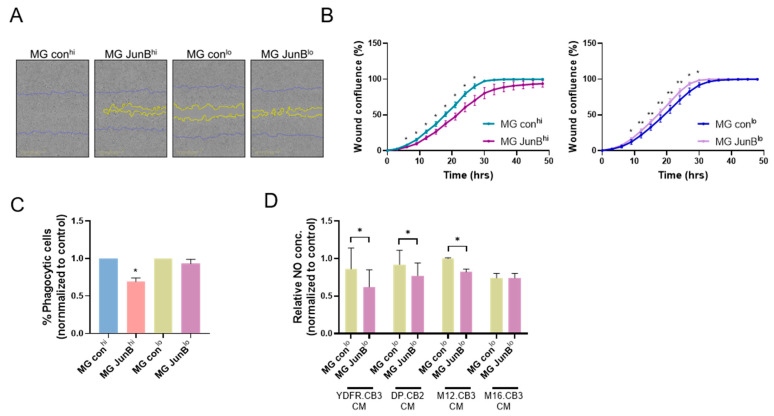
JunB impacts the functional phenotype of microglia cells. (**A**,**B**) JunB^hi^ and JunB^lo^ microglia cells and their controls were seeded in a monolayer. Upon confluence, a scratch was performed in each well and cells were allowed to migrate. Images were acquired every 3 h for 48 h using Incucyte S3. (**A**) Representative images of wound-healing assay at 27 h. Scale bar: 500 μm. (**B**) Analysis of the mean + SEM of wound confluence (%) was obtained from the Incucyte S3 software (v2019A). * *p* < 0.05, ** *p* < 0.01. (**C**) Phagocytosis of fluorescent beads as determined by flow cytometry analysis. JunB^hi^ and JunB^lo^ microglia cells and their controls were incubated with fluorescent latex beads for 4 h. The bars represent the mean percentage of phagocytic (fluorescence positive) cells (normalized to control) + SEM, * *p* < 0.05. (**D**) JunB^hi^ and JunB^lo^ microglia cells and their controls were seeded in 96-well plate and treated with concentrated MCM (×30) for 24 h, after which the nitric oxide (NO) concentration in the medium was measured by Griess reagent, as well as the NO concentration in the concentrated MCM as control. The bars represent the relative NO concentration (NO concentration measured 24 h after treatment divided by NO concentration of the initial concentrated MCM) + SEM, * *p* < 0.05.

First, proliferation assays employing XTT-based assays demonstrated that there were no differences between the viability of JunB^hi^ or JunB^lo^ microglia cells and their corresponding controls (Supplementary Figure S1B). Then, wound-healing assays demonstrated that JunB^hi^ microglia cells migrated slower and JunB^lo^ microglia cells migrated faster than their corresponding control cells (Figure 4A,B). Since there was no difference in the proliferation rate between JunB^hi^ and JunB^lo^ microglia cells and their controls (see above), we conclude that the two microglia populations, that differ in JunB expression, differ in their migration rate.

We next tested the phagocytic activity of microglia cells by measuring the engulfment of latex beads after 4 h of incubation, followed by flow cytometry analysis. Figure 4C demonstrates that JunB^hi^ microglia cells exhibit a lower phagocytic activity than control cells. JunB^lo^ microglia cells showed no difference in phagocytic activity compared to controls.

The results described above demonstrate that the two microglia populations, differing in JunB expression, differ in their potential to promote pro-tumor or anti-tumor effects.

#### 3.2.6. JunB^lo^ Cells Exhibit Increased Consumption of Melanoma-Derived Nitric Oxide (NO)

Macrophages synthesize and secrete the cytotoxic molecule NO, that is able to kill pathogens as well as induce apoptosis and necrosis of cells [51]. Melanoma cells also produce NO which, conversely, contributes to their survival [52].

We measured NO production by melanoma-exposed microglia cells expressing low levels of JunB. JunB^lo^ and control cells were stimulated with MCM from four MBM cell lines (concentrated x30) for 24 h, after which NO concentration was measured in the supernatants collected from microglia cultures. NO concentration was also measured in MCM to serve as baseline control. Indeed, all MBM cells produced NO. No significant change was observed between MCM and control microglia supernatants (suggesting a significant consumption of NO did not occur), however, NO levels were lower in JunB^lo^ microglia supernatants compared to NO levels measured in control microglia supernatants, except in the M16.CB3 CM-treated microglia (Figure 4D), indicating that melanoma-derived NO might have been taken up by JunB^lo^ microglia, as shown in macrophages [53], and this in turn may restrain the NO-dependent proliferative effect on melanoma cells.

### 3.3. The Involvement of Melanoma-Associated Microglia Cells Expressing High or Low JunB Levels in the Progression of Brain-Metastasizing Melanoma

#### 3.3.1. Factors Released from JunB^lo^ Microglia Restrain the Proliferation and Migration of MBM Cells

In the next series of experiments, we asked if JunB-expressing microglia are involved in regulating the malignant phenotype of neighboring melanoma brain metastases. To this end, we evaluated the functional impact of factors released from JunB^hi^ and JunB^lo^ microglia cells on the viability and migratory capacity of MBM.

**Figure 5:**
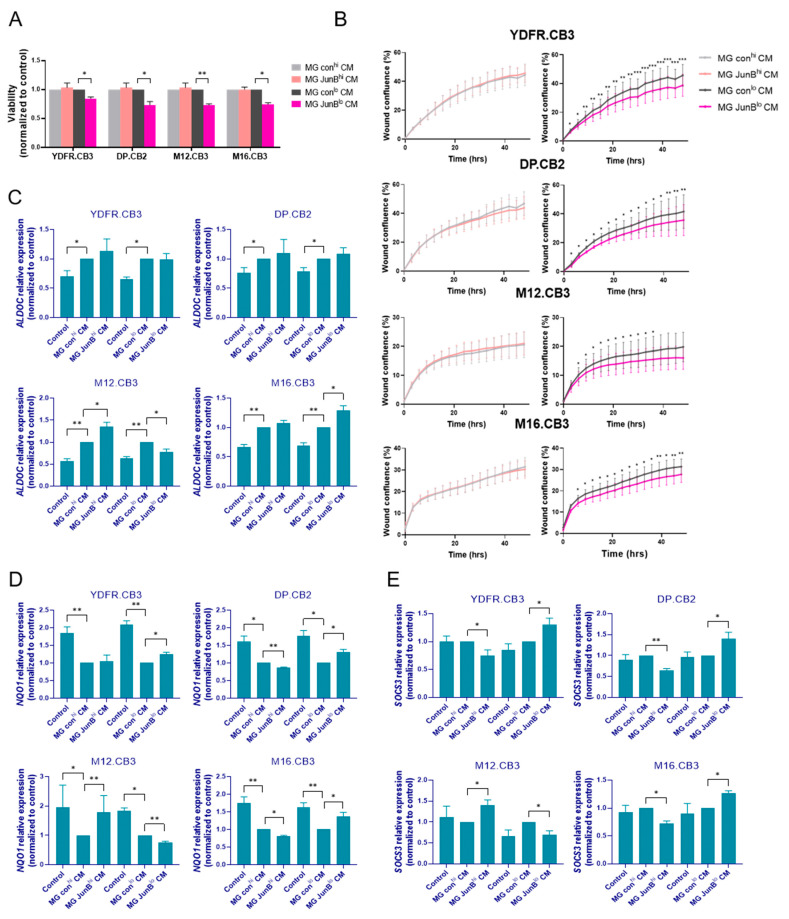

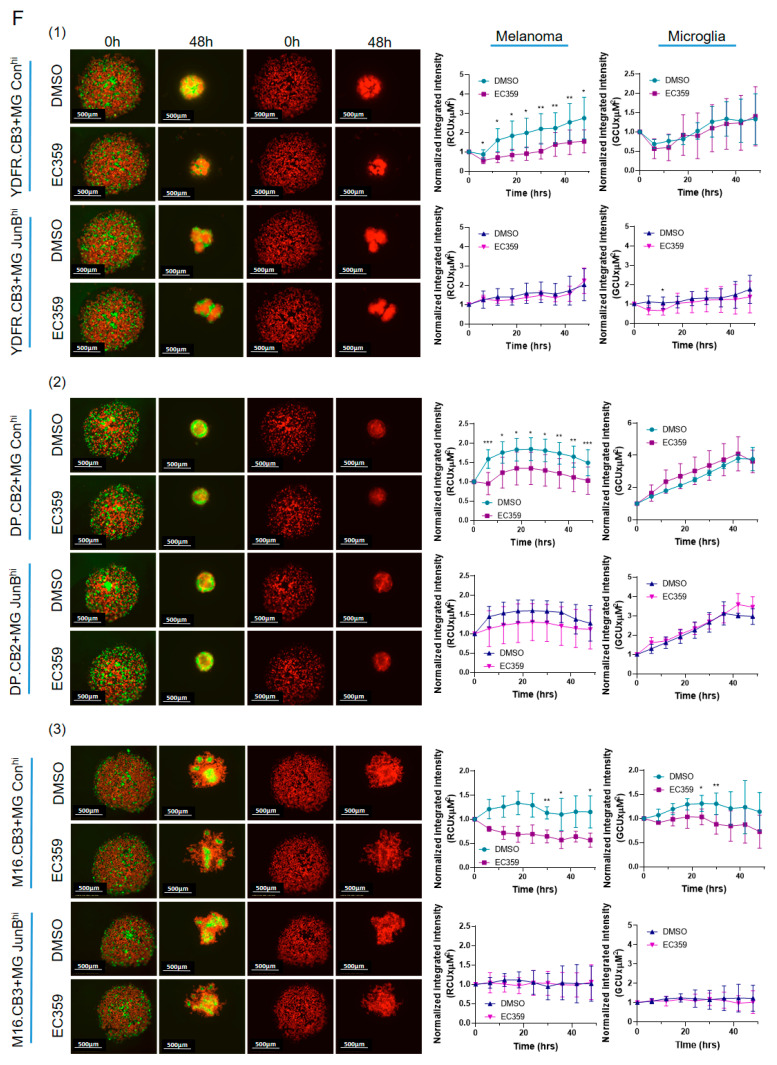
Microglial JunB promotes the malignant phenotype of MBM cells. (**A**) Viability of MBM cells treated with CM of JunB^hi^ and JunB^lo^ microglia cells and their controls for 48 h was measured by XTT. The bars represent the mean viability of melanoma cells treated with CM of JunB^hi^ and JunB^lo^ microglia cells normalized to cells treated with CM of control microglia + SEM. * *p* < 0.05, ** *p* < 0.01. (**B**) MBM cells were seeded in a monolayer. Upon confluence, cells were treated with mitomycin C for 3 h, then a scratch was performed in each well, CM of JunB^hi^, JunB^lo^, and matching control microglia cells was added, and cells were allowed to migrate. Images were acquired every 3 h for 48 h using Incucyte S3. Analysis of the mean + SEM of wound confluence (%) was obtained from the Incucyte S3 software. * *p* < 0.05, ** *p* < 0.01, *** *p* < 0.005. (**C**–**E**) The relative expression of ALDOC (**C**), NQO1 (**D**), and SOCS3 (**E**) mRNA in MBM cells treated with starvation medium or with CM of JunB^hi^ and JunB^lo^ microglia cells and their controls for 24 h was detected by RT-qPCR. RS9 was used for gene expression normalization. The bars represent mean expression (normalized to MG con^hi^ or MG con^lo^ cells) + SEM, * *p* < 0.05, ** *p* < 0.01. (**F**, 1–3) Spheroid formation assay of mCherry-labeled MBM cells and GFP-labeled microglia cells (1:1) with EC359 or DMSO, imaged for 48 h using the IncuCyte system. Representative images of the wells at the beginning and end point of the experiment are presented. Mean mCherry integrated intensity (RCU×µM^2^) and GFP integrated intensity (GCU×µM^2^) ± SEM are presented in the graphs. * *p* < 0.05, ** *p* < 0.01, *** *p* < 0.005. Experiments were performed at least three times, in 4–6 replicates. Scale bar: 500 μm.

The four MBM cell lines utilized in this study were exposed for 48 h to factors released from JunB^hi^ or JunB^lo^ microglia cells and from their respective control cells. The results showed that factors released from JunB^lo^ microglia cells decreased the proliferation rate of the four melanomas, while factors released from JunB^hi^ microglia cells did not affect the proliferation rate of these melanoma cells (Figure 5A).

PI and annexin V staining did not indicate the occurrence of apoptotic or necrotic cell death of the melanoma cells exposed to factors released from JunB^lo^ microglia cells (Supplementary Figure S1C), therefore, we tentatively conclude that these factors reduced the proliferation of the melanoma cells by an as yet unidentified cytostatic mechanism. Alternatively, such factors may be deficient in mitogenic activity required for cellular proliferation.

In addition to proliferation, migratory and invasive capacity of cancer cells are key factors in their progression to metastasis [54]. To determine if microglia expressing high JunB levels are involved in regulating the migratory capacity of melanoma cells, we performed wound-healing assays of MBM cells in the presence of JunB^hi^ or JunB^lo^ microglia-derived secreted factors. MBM cells were treated with mitomycin C 3 h prior to treatment to avoid bias resulting from their proliferation. Whereas factors released from JunB^hi^ microglia cells did not affect the migration of melanoma cells, factors released from JunB^lo^ microglia cells reduced the migratory capacity of melanoma cells (Figure 5B).

#### 3.3.2. Factors Released from JunB^hi^ and JunB^lo^ Microglia Regulate ALDOC, NQO1, and SOCS3 Gene Expression in MBM Cells

The glycolytic pathway is intimately linked to cancer due to the high metabolic demand of cancer cells [55]. Previously, we showed that the glycolytic enzyme aldolase C played opposing roles in the formation and maintenance of brain metastasis in different melanomas [21]. Aldolase, fructose-bisphosphate C (ALDOC), and NAD(P)H quinone oxidoreductase 1 (NQO1) were shown to be upregulated and downregulated, respectively, in microglia CM-treated MBM cells [5]. In contrast to its immunosuppressive effect in macrophages and microglia cells, SOCS3 has a protective, anti-tumor role in melanoma [56].

qPCR analysis demonstrated that factors released from JunB^hi^ microglia cells increased ALDOC expression in M12.CB3 and JunB^lo^-secreted factors had the opposite effect in these cells. However, factors released from JunB^lo^ cells increased ALDOC expression in M16.B3 cells (Figure 5C).

Exposure to factors secreted from JunB^hi^ cells resulted in decreased NQO1 expression in DP.CB2 and M16.CB3. NQO1 expression was increased in three of the MBM cells following exposure to factors secreted from JunB^lo^ cells, while in M12.CB3 NQO1 expression was decreased (Figure 5D).

The expression of SOCS3 in three of the MBM cells was decreased following exposure to JunB^hi^-secreted factors and increased following exposure to JunB^lo^-secreted factors, with the exception of M12.CB3 cells, that exhibited an opposite trend (Figure 5E).

These findings demonstrate that microglia cells expressing different JunB levels secrete a different set of cytokines, differently affecting the expression of malignancy-related genes such as ALDOC, NQO1, and SOCS3 in MBM cells. In general, JunB^hi^-secreted factors promoted a molecular alteration supporting malignancy progression (in three out of the four cell lines), while JunB^lo^-secreted factors promoted a molecular alteration restraining malignancy progression (in three out of the four cell lines).

The heterogenous effect on gene expression in a cell line-dependent manner suggests that different MBM cells may utilize different immune escape or survival strategies in metastasis progression.

#### 3.3.3. Microglial JunB Positively Regulates Melanoma Cell Proliferation in Melanoma–Microglia Spheroids via LIFR

As demonstrated above, melanoma-derived LIF reprogrammed the molecular and functional phenotype of LIFR-expressing microglia cells.

Three-dimensional (3D) cultures of cancer cells and cells in their microenvironment recapitulate the in vivo cell–cell contact and interaction [57].

In the next set of experiments, we asked if the LIF/LIFR interaction is directly involved in mediating microglia-supported MBM cell proliferation and if this putative effect is JunB-dependent. mCherry-labeled YDFR.CB3, DP.CB2, and M16.CB3 were co-cultured with GFP-labeled JunB^hi^ or con^hi^ microglia cells to form spheroids, which were treated with LIFR inhibitor (EC359) or DMSO as a control. M12.CB3 cells do not form spheroids and were thus excluded from this experiment. Analysis of the integrated intensity showed a significant decrease in mCherry intensity in all three melanoma cell lines co-cultured with GFP-labeled con^hi^ microglia cells following treatment with EC359, compared to DMSO (control) (Figure 5F). Spheroids of mCherry-labeled melanoma cells and GFP-labeled JunB^hi^ microglia cells showed no decrease in mCherry intensity when treated with EC359, compared to DMSO.

These results suggest that the proliferation of MBM in MBM–microglia co-cultures is regulated by microglial JunB and that the expression of which is regulated by LIFR.

## 4. Discussion

The progression of cancer cells that seed a secondary organ towards metastasis depends on a collaborative crosstalk between these cells and organ-specific microenvironmental components of this organ [58].

It is established that TAMs promote cancer progression by enhancing tumor cell proliferation, migration, angiogenesis, and immune suppression [59]. Previous studies indicated that microglia cells take part in the formation and maintenance of brain metastasis [60]. Previous work from our lab confirmed these findings by demonstrating that human melanoma cells and microglia reprogram each other’s phenotype, thereby promoting melanoma towards brain metastasis. Treating microglia with factors present in the conditioned medium of brain-metastasizing melanoma cells resulted in a significant remodeling of the phenotype and functions of microglia cells [6]. Reciprocally, microglia advanced the malignancy of melanoma cells by stimulating various pro-metastatic properties in MBM cells, for example, by boosting their proliferation and migration capacities [5].

In the present study, we showed that a short-term in vitro exposure of microglia to MCM resulted in a significant increase in the expression of the transcription factor JunB in these microglial cells. Supporting these results, unpublished data of single-cell RNA-seq of microglia derived from MBM-bearing mice demonstrated an upregulated expression of JunB in MBM-associated microglia, compared to microglia from healthy mice.

We showed that two members of the IL-6 family, LIF and OSM, induce JunB upregulation in microglia, a finding that was previously reported with respect to LIF-treated human carcinoma cells and to OSM-treated primary chondrocytes [61,62]. Whereas MBM cells secreted LIF, we did not detect soluble OSM in MCM. Therefore, we focused on the LIF/LIFR axis and demonstrated (in three out or four MBM cell lines used in this study) that microglial LIFR activation by LIF in melanoma-exposed microglia induces downstream signaling of the JAK/STAT3 pathway, leading to JunB upregulation.

The functions of JunB in macrophages and microglia have not been thoroughly studied thus far. However, recent papers demonstrated its role in these cells in several pathologies. JunB expression in bladder cancer-associated immunosuppressive cells was previously reported by Chen et al. [13]. JunB was identified as a central regulator of the immunosuppressive, non-inflamed TME, as it negatively correlated with antigen presentation by MHC molecules, immune cell recruitment, anti-cancer immune response, and immune checkpoint inhibitors, thereby contributing to an unfavorable prognosis of tumor patients. Secondly, JunB mediated the release of inflammatory factors in dorsal root ganglion macrophages, promoting neuropathic pain [63]. In addition, microglial JunB inhibited inflammation and apoptosis during ischemia/reperfusion in brain injury [64].

We have shown that the expression pattern of JunB in MCM-treated microglia differs from that of untreated microglia. Whereas the latter cells are rather homogeneous with respect to JunB expression, there was an expansion of cells expressing relatively high levels of JunB in MCM-treated microglia.

These findings served as a rationale for the generation of stable microglia variants expressing either relatively high JunB (JunB^hi^) or relatively low JunB (JunB^lo^) levels.

Previous reports indicated that subpopulations of microglia cells may express diverse, often opposing phenotypes and functions [65]. Such heterogeneity with respect to JunB expression was reported, for example, in Alzheimer’s disease, where JunB was expressed only in a specific population of an activated, highly phagocytic, stage 1 disease-associated microglia (DAM-1) [18].

The above results demonstrated that microglia cells expressing high and low JunB levels behaved differently.

In general, microglia expressing high JunB levels presented a pro-tumorigenic phenotype. RPPA analysis indicated that JunB^hi^ cells expressed higher levels of CD44 than control microglia. CD44 negatively regulates TLR signaling and is involved in resolution of inflammation [66]. Additionally, JunB^hi^ microglia expressed lower levels of phosphorylated c-Jun (Ser73) than control microglia. JunB and c-Jun have opposing functions [67], as c-Jun mediates pro-inflammatory responses in macrophages [68,69].

JunB^hi^ cells expressed significantly higher levels of the immunosuppressive molecules SOCS3 and PD-L1. SOCS3 in myeloid cells is mostly linked to cancer-promoting anti-inflammatory macrophages and repression of the pro-inflammatory phenotype [45,70]. We have recently shown that melanoma-mediated SOCS3 upregulation in microglia promoted tumor-supporting properties in the latter cells [6]. Also, loss of SOCS3 in macrophages prolonged the survival of glioma-bearing mice [70]. PD-L1 expressed by microglia regulates immune responses by interacting with T-cell-expressed PD-1, thereby limiting T-cell activation [71].

Functionally, JunB^hi^ microglia cells exerted reduced phagocytosis and migration, perhaps via the downregulation of SERPINE1, that is known to inhibit microglial phagocytic activity and has a dual role in the regulation of cell migration depending on its binding partners [48].

Factors secreted by these cells downregulated NQO1 and SOCS3 expression in some of the melanomas. Both NQO1 and SOCS3 are associated with lower malignancy grade in melanoma [72,73]. Therefore, their down-regulation may contribute to MBM progression.

Examining the involvement of LIF/LIFR interaction between MBM cells and microglia, we found that inhibition of LIFR presumably abrogated JunB upregulation in microglia following interaction with melanoma cells and, consequently, by repression of the transcriptional regulation induced by JunB, modified microglia phenotype so that it limited melanoma cell proliferation. The 3D co-cultures of melanoma with JunB-overexpressing microglia protected melanoma cells from the growth-restraining effect mediated by LIFR inhibition, further confirming that LIFR positively regulates MBM cell proliferation predominantly by microglial JunB.

Opposite to that, microglia expressing low JunB levels presented an anti-tumorigenic phenotype. JunB^lo^ cells were highly migratory and consumed lower levels of NO, thereby depriving melanoma cells of NO-induced proliferation [53].

These cells expressed higher levels of Iba-1, which is correlated with microglial activation [74], and higher levels of CD150, which manifests both pro- and anti-malignant functions. On the one hand, it is an M2 marker with immune regulatory functions [75], however, there are data showing that CD150 may also function as a cytotoxic anti-cancer molecule [76]. Furthermore, despite the possible immune regulatory activity of CD150, JunB^lo^ microglia present other pro-inflammatory molecular and functional traits that may govern the effect of CD150.

For instance, based on RPPA results, the expression of Ras homolog enriched in brain (Rheb), which upregulates inflammatory genes [77], was higher in JunB^lo^ cells than in control microglia. JunB^lo^ microglia also expressed higher levels of c-Jun activation domain-binding protein-1 (JAB1) than control. This protein interacts with the activation domain of c-Jun and stabilizes its complexes on AP-1 binding sites, thereby enhancing inflammatory responses [78].

Further support for the tumor-restraining functions of JunB^lo^ microglia was provided by findings that factors secreted from JunB^lo^ microglia significantly reduced two malignancy hallmarks of cancer cells, namely proliferation and migration [79], of all four MBM cell lines.

Factors secreted by these cells upregulated NQO1 and SOCS3 expression in melanoma. The decreased proliferation and migration of YDFR.CB3, DP.CB2, and M16.CB3 melanoma cells exposed to factors released from JunB^lo^ microglia may have been instigated by an increased transcription of SOCS3, a negative feedback regulator of the STAT3 signaling pathway [80], mediating melanoma cell proliferation and migration [81,82]. Thus, an increase in SOCS3 transcription in MBM exposed to factors released from JunB^lo^ microglia cells can inhibit the STAT3-mediated pro-tumor effects.

Although the transcriptional response of M12.CB3 cells to factors secreted by JunB^lo^ microglia was opposed to that of the other three melanoma cells, these factors affected the proliferation and migration of M12.CB3 cells similarly. It is possible that the proliferation and migration of M12.CB3 cells are attenuated by JunB^lo^ microglia-derived factors through a reduction in ALDOC transcription, as we previously demonstrated that microglia-derived factors increased the proliferation of M12.CB3 cells overexpressing the glycolytic enzyme ALDOC [21], which is essential for cancer cell proliferation and migration [83,84].

The fact that microglial JunB may exert the same effects on the malignancy phenotype of different melanoma cell lines through different mechanisms, highlights again, as we have already demonstrated, the issue of intertumor heterogeneity with respect to tumor–microenvironment interactions [6,21,85,86]. As MBM cells that originate from different patients differ in their genetic expression and mutation profile, these genetic differences do not only influence tumor cell phenotype but also the consequences of interactions with the surrounding microenvironment. The tumor microenvironment affects the transcription of genes via distinct, cell-specific signaling pathways activated in tumor cells by extracellular factors in the microenvironment [87] and vice versa. For instance, lung adenocarcinoma cells carrying a mutation in *EGFR* recruit fewer PD-L1^+^/CD8^+^ tumor-infiltrating lymphocytes compared to *TP53*- and *KRAS*-mutated cells. This might explain the unfavorable response to PD-1 blockade in patients carrying the EGFR mutation compared to patients carrying the *TP53*/*KRAS* mutation [88]. This highlights the need for caution when drawing general conclusions based on in vitro or in vivo results employing single or a very few cell lines.

Put together, melanoma-derived factors segregate microglia into cell populations expressing high or low (basal level) JunB. Cells expressing high JunB levels were generally polarized into anti-inflammatory, immunosuppressive, and pro-tumor microglia, while cells expressing low JunB levels were generally polarized into pro-inflammatory, anti-tumor microglia.

Our findings provide mechanistic evidence suggesting microglial JunB, as a switch molecule, induced by the neighboring melanoma cells to harness microglia in their favor, and targeting this specific microglia subpopulation may serve as a therapy for brain-metastasizing melanoma.

## 5. Conclusions

Microglia exposure to factors secreted by MBM cells, mainly LIF, resulted in the generation of heterogenous populations of microglia expressing high or low JunB levels. A population of microglia highly expressing JunB exhibited a pro-tumor phenotype, contributing to an immunosuppressive microenvironment, with a modified secretome that promoted melanoma gene expression alterations. The population of microglia expressing low levels of JunB, on the other hand, exhibited an anti-tumor phenotype, attenuating melanoma progression.

Overall, we identified microglia highly expressing JunB as a determinant of the progression of brain-metastasizing melanoma cells. As such, targeting this microenvironmental molecule may serve as a novel theranostic target.

## Data Availability

Data were submitted to the GEO database. Accession numbers will be provided when available.

## Supplementary Materials

The following supporting information can be downloaded at: https://www.mdpi.com/article/10.3390/cancers15204979/s1, Figure S1: Characterization of JunB^hi^ and JunB^lo^ microglia cells and the effect of their secreted factors on MBM cells; Table S1: List of antibodies utilized in the study; Table S2: Enriched biological processes of the differentially expressed genes obtained by DAVID.
