# Supplementary material for: Heterogeneity in the Metastatic Microenvironment: JunB-Expressing Microglia Cells as Potential Drivers of Melanoma Brain Metastasis Progression
Source: Cancers (Basel). 2023 Oct 13;15(20):4979. doi: 10.3390/cancers15204979 (PMC10605008; doi:10.3390/cancers15204979)
Supplement: Supplementary file 1 [file cancers-15-04979-s001.zip › Supplementary Table S1.pdf]

Supplementary Table S1. List of antibodies utilized in the study.

Antibody	Catalogue no.	Origin[1]	Application	Concentration	Manufacturer details
Polyclonal Anti-AIF-1/Iba1	NB100-1028	Goat	IHC (frozen)<br>FACS	1:70<br>1:50	Novus Biologicals, Centennial, CO, USA
Polyclonal Anti-β-tubulin	ab6046	Rb	WB	1:1000	Abcam, Cambridge, MA, USA
Monoclonal Anti-CD14, REAfinity™	130-110-578	Human	FACS	1:50	from Miltenyi Biotec, Bergisch Gladbach, Germany
Monoclonal Anti-CD16, REAfinity™	130-113-951	Human	FACS	1:50	from Miltenyi Biotec, Bergisch Gladbach, Germany
Monoclonal Anti-CD150 (SLAM), REAfinity™	130-099-722	Human	FACS	1:50	from Miltenyi Biotec, Bergisch Gladbach, Germany
Monoclonal Anti-CD163, REAfinity™	130-112-287	Human	FACS	1:50	from Miltenyi Biotec, Bergisch Gladbach, Germany
Monoclonal Anti-gp130	sc-9994	Ms	FACS	1:20	Sana Cruz Biotechnology, Inc., Dallas, TX, USA
Monoclonal Anti-JunB	3753	Rb	IHC (frozen)<br>FACS	1:100<br>1:50	Cell Signaling Technology, Inc., Danvers, MA, USA
Polyclonal Anti-LIFRβ	22779-1-AP	Rb	FACS	1:50	Proteintech, Rosemont, IL, USA
Multiclonal Anti-melanoma (targets HMB45, MART-1 and Tyrosinase)	ab733	Ms	IHC (frozen)	1:100	Abcam, Cambridge, MA, USA
Monoclonal Anti-PD-L1	393602	Ms	FACS	1:50	BioLegend, San Diego, CA, USA
Monoclonal Anti-phospho-STAT3 (Tyr705)	9145	Rb	WB	1:1000	Cell Signaling Technology, Inc., Danvers, MA, USA
Monoclonal Anti-Stat3	9139	Ms	WB	1:500	Cell Signaling Technology, Inc., Danvers, MA, USA

[1]Ms: mouse, Rb: rabbit
