# Supplementary figures and images for: Heterogeneity in the Metastatic Microenvironment: JunB-Expressing Microglia Cells as Potential Drivers of Melanoma Brain Metastasis Progression
Source: Cancers (Basel). 2023 Oct 13;15(20):4979. doi: 10.3390/cancers15204979 (PMC10605008; doi:10.3390/cancers15204979)

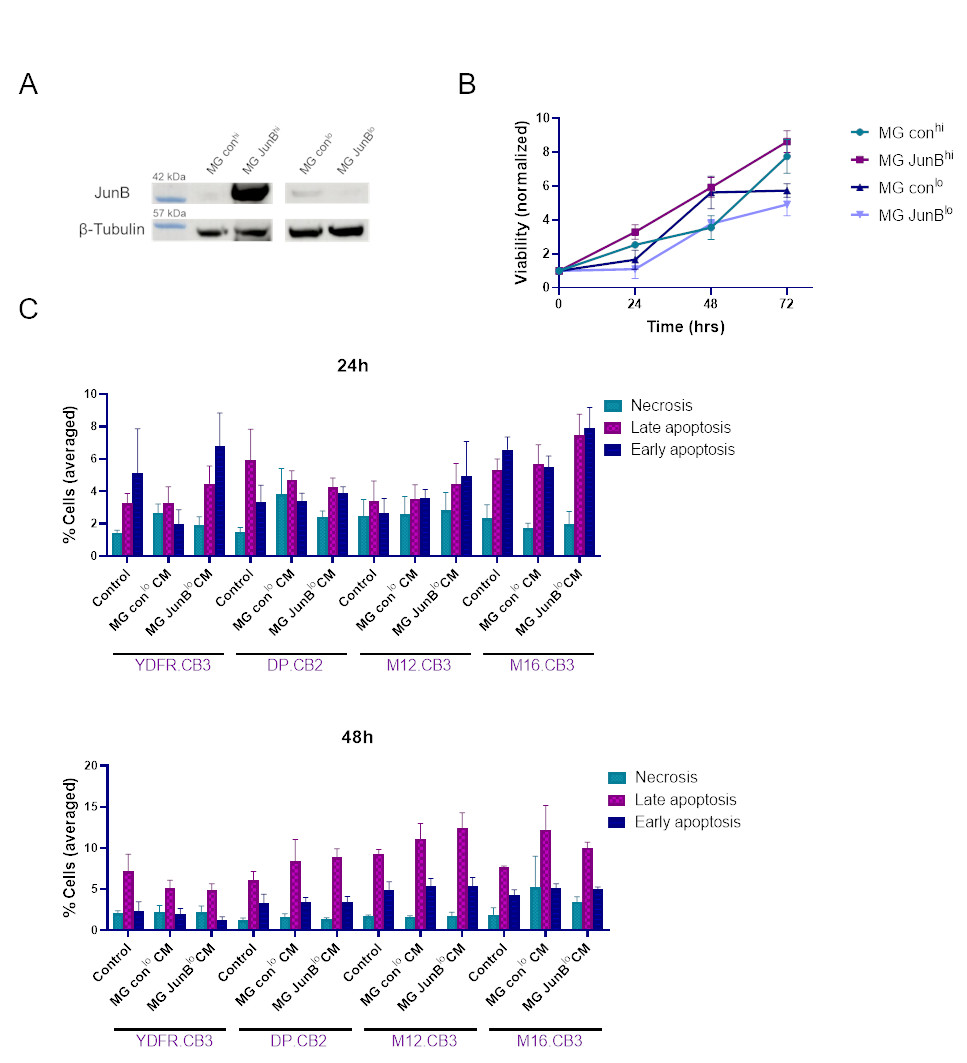

Supplement: Supplementary file 1 [file cancers-15-04979-s001.zip › Supp. Fig. S1.jpg]
